# Supplementary material for: Learning noise-induced transitions by multi-scaling reservoir computing
Source: Nat Commun. 2024 Aug 3;15:6584. doi: 10.1038/s41467-024-50905-w (PMC11297999; doi:10.1038/s41467-024-50905-w)
Supplement: Supplementary file 1 — SUPPLEMENTARY INFO [file 41467_2024_50905_MOESM1_ESM.pdf]

Supplementary Information: Learning noise-induced transitions by multi-scaling reservoir computing

Zequn Lin,[1,2,3,4,*] Zhaofan Lu,[2,*] Zengru Di,[2] and Ying Tang[1,5,2,†]

[1] *Institute of Fundamental and Frontier Sciences, University of Electronic Science and Technology of China, Chengdu 611731, China*
[2] *Department of Systems Science, Faculty of Arts and Sciences, Beijing Normal University, Zhuhai 519087, China*
[3] *Center for Interdisciplinary Studies, Westlake University, Hangzhou 310024, China*
[4] *School of Science, Westlake University, Hangzhou, 310024, China*
[5] *Key Laboratory of Quantum Physics and Photonic Quantum Information, Ministry of Education, University of Electronic Science and Technology of China, Chengdu 611731, China*

CONTENTS

I. Supplementary note 2
  A. Applying previous approaches to learn stochastic transitions 2
    1. Results of SINDy 2
    2. Results of RNN 2
    3. Preprocessing data with noise by filters 2
    4. Results of FORCE learning 3
    5. Comparisons of SINDy and FORCE learning on protein folding data 3
  B. Effectiveness of present method in mixed time series 3
  C. Power spectral density as an indicator for accurate training 4
  D. The robustness of the results under a range of hyperparameters 4
  E. Assessing the impact of training set length on performance 4
  F. More Examples 5
    1. A one-dimensional tilted bistable gradient system 5
    2. A two-dimensional bistable gradient system 5
    3. A two-dimensional tilted bistable gradient system 5
    4. A two-dimensional tilted bistable non-gradient system 5
    5. A two-dimensional tristable system 6
    6. A bistable gradient system with high-dimensional colored noise 6

II. Supplementary figures 8

III. Supplementary tables 28

Supplementary References 30

* These authors contributed equally
† jamestang23@gmail.com

I. SUPPLEMENTARY NOTE

In the Supplementary Note, we first apply conventional approaches to noise-induced transitions, including Sparse Identification of Nonlinear dynamics (SINDy) [1–3], recurrent neural network (RNN) [4, 5], filters (Kalman filter [6] and Savizky-Golay filter [7]), and First-Order, Reduced, and Controlled Error (FORCE) learning [8, 9]. Second, we analyze the frequencies of the deterministic part and the noise using fast Fourier transform (FFT) to investigate the effectiveness of the present method when frequencies are mixed. Third, we provide a protocol to search for the hyperparameters by using power spectral density, and also investigate the robustness of reservoir computing (RC) outcomes under the hyperparameters α and β. Next, we decrease the length of the training set to study its effect on training. We further apply the present method to more systems to supplement the main text, including a 1D tilted gradient system, a 2D gradient system, 2D tilted gradient and non-gradient systems, a 2D tristable system, and a 1D bistable system with Lorenz-96 noise.

A. Applying previous approaches to learn stochastic transitions

1. *Results of SINDy*

SINDy is widely applied to identify the underlying dynamical system and to learn noise distribution from time series [1, 3]. It captures the nonlinear dynamics with sparsity-promoting techniques and automatic differentiation. However, it remains uncertain whether SINDy can effectively learn noise-induced transitions. To assess the effectiveness of SINDy, we apply two types: SINDy-2016 [1] and SINDy-2021 [3], to the Example 1 of the main text.

First, we utilize SINDy-2016 to learn the data with noise. We generate a time series from Eq. (9) of the main text as the training data and utilize SINDy-2016 to fit it. In detail, we select the polynomial functions as the basic and the order is 3 (Eq. (9) is a polynomial function). The parameter threshold represents the minimum value for the coefficients of the fitting function. As in Supplementary FIG. 2(a, b), the outcomes of SINDy-2016 (green lines) are distinctly different from the test data (coral lines). These results demonstrate that SINDy-2016 does not directly capture the noise-induced transitions. The identifications are in Supplementary Table I Set 1-2.

The SINDy-2016 is not designed for data with noise, whereas the SINDy-2021 can identify the dynamics and separate noise distribution from data with noise. We apply SINDy-2021 to capture the slow-scale dynamics and generate the series with the separated noise distribution. In Supplementary FIG. 2(c), we compare the generated series (green line) with the actual data (coral line), the identification result is in Supplementary Table I Set 3, and evaluate their transition time in $10000\delta t$ (Supplementary FIG. 2(d)). The evaluation of the true and predicted data presents significant difference. As we adjust the value of threshold, the results (Supplementary Table I Set 4) also lack accuracy (Supplementary FIG. 2(e, f)). These results show that SINDy-2021 also does not deal with stochastic transitions from data with noise. Furthermore, the computational time of SINDy-2021 is greater than RC (Supplementary Table II).

2. *Results of RNN*

We select a bistable system with white noise generated from Eq. (9) of the main text to demonstrate whether RNN [4] can predict stochastic transitions. We apply RNN based on the Python library, Keras [5]. We utilize a variant of RNN, Long Short-Term Memory (LSTM), with 10 iterations (epochs). The number of neurons (units) and batch size are adjusted to tune the model, and results are illustrated in Supplementary FIG. 3. The results, significantly different from the actual data, demonstrate the challenge of learning noise-induced transitions. Moreover, the computational cost of RNN is higher than RC (Supplementary Table II).

3. *Preprocessing data with noise by filters*

We apply the Kalman filter [6] and the Savitzky-Golay filter [7] to preprocess the training data to address the limitation that SINDy-2016 is not designed for data with noise (Supplementary FIG. 2(a, b)). By inputting the filtered data to SINDy-2016 for learning, the output functions of SINDy-2016 are in Supplementary Table III. As a comparison, we also train RC using filtered data.

Supplementary FIG. 4(a-d) present the results with the Kalman filter, the measurement noise covariance matrix Q represents the uncertainty or the strength of the noise. The SINDy-2016 and RC are unable to learn the filtered time series. Supplementary FIG. 4(e-h) show the outcomes with the Savizky-Golay filter, the window length W is

the fitting length. A larger W leads to a smoother output, and the polynomial order P controls the polynomial order of the filter fit. The results do not show stochastic transitions.

4. Results of FORCE learning

FORCE learning has been shown to be able to generate time series that are not generated from explicit low-dimensional dynamical systems, especially on systems with noisy and chaotic systems [8]. We use the tension Python package for FORCE learning [9]. Initially, we test the full-FORCE model, a basic FORCE learning architecture [8], for the one-dimensional bistable system Eq. (9) of the main text. We employ the same hyperparameters as in Figure 4 of [9]. As shown in Supplementary FIG. 5(a), the prediction after 10 epochs of full-FORCE training displays larger fluctuations than the training data, especially at longer time. More importantly, it takes around 10 times more computational time than the present method (Supplementary FIG. 5(b)). In all these time comparisons, each method uses a fixed set of hyperparameters, excluding the time required for hyperparameter tuning.

Next, we apply the FORCE-trained spiking neuron model, using the same hyperparameter as those in the learning Lorenz system (Figure 6 in [9]). The key hyperparameter is the update interval, which determines how often FORCE updates are applied. We observe that with an interval of 50, as chosen in Figure 6 of [9], the transition time of true and predicted data do not match (Supplementary FIG. 6(a)). We also attempt to search for better values of this hyperparameter. With update interval of 10, the predicted transition time becomes more accurate (Supplementary FIG. 6(b)), with the trajectory shown in Supplementary FIG. 6(d), and is less accurate with a further decrease in the update interval to 1 (Supplementary FIG. 6(c)). Despite its match on the transition time for the specific choice of the hyperparameters, the computational time is always a few times more than ours.

Additionally, we apply the spiking neuron model to protein folding data with the same training length as in Example 4 of the main text (T_{train}: 25000, 7500, 6000 time steps). With the same hyperparameters in Supplementary FIG. 6(b), none of these predictions capture the stochastic transition of this real data (Supplementary FIG. 7).

5. Comparisons of SINDy and FORCE learning on protein folding data

We compare the present method with the SINDy-2021 and FORCE learning methods using training sets of 7500 time steps and double that amount to further clarify the performance of the present method on experimental data (Example 4 of the main text). The present method accurately captures the average downward transition time and its distribution with small distance values (Supplementary FIG. 8(a, b)), indicating a good match between prediction and true data. In contrast, SINDy-2021 (Supplementary FIG. 8(c, d)) and FORCE learning (Supplementary FIG. 8(e, f)) exhibit larger distance values, failing to accurately capture the transition time. It demonstrates that the present method achieves significantly higher accuracy than these state-of-the-art methods.

B. Effectiveness of present method in mixed time series

A time series with stochastic transitions can contain multiple frequencies, and there can be various scenarios in which the noise signal are mixed into the frequency distribution of the deterministic dynamics. To assess the effectiveness of the present method in this case, we utilize FFT based on the Python library Numpy[10] to analyze the frequencies of the time series.

We analyze the frequencies of data with white noise (Example 1 of the main text). Due to the mathematical properties of white noise, the FFT result shows mostly equal intensity at different frequencies except frequencies of deterministic dynamics (Supplementary FIG. 20(a)). Next, we examine the frequencies from Eqs. (10)-(13) of the main text (FIG. 3(b) of the main text), a bistable system with colored noise. We find that the frequency distribution is similar to Supplementary FIG. 20(a), it indicates that the frequencies of Lorenz noise and deterministic dynamics are mixed (Supplementary FIG. 20(b)), and the prediction is shown in FIG. 3 of the main text. These results illustrate that the present method is effective.

To further demonstrate the effectiveness of the current method in handling mixed frequency distributions, we adjust the parameters ϵ and ψ in the Eqs. (11)-(13) of the main text. As the values $\epsilon = 1.4, \psi = 0.13$, the time series, and the FFT result are presented in Supplementary FIG. 20(c), the frequency distribution appears to be mixed. By repeating the process as in the Example 2 of the main text, the results display the effectiveness of the present method when the frequencies are mixed (Supplementary FIG. 20(d, e)).

C. Power spectral density as an indicator for accurate training

The power spectral density (PSD) and the autocorrelation function of a series form a Fourier transform pair. It can help search for the hyperparameters when learning a stochastic series. The present manual tuning method prefers to have prior information on the number and locations of the basins. For many datasets, such information can be roughly estimated directly from the time series, such as by segmenting the time series between large jumps and calculating the mean value of each segment. This procedure is used in analyzing the experimental data (Figure 4 of [11]). When it is challenging to identify the locations of basins directly from stochastic data, we find that the PSD quantifies the accuracy of learning basins. When deterministic dynamics is accurately predicted, the power spectrum of the predicted stochastic time series better matches that of the training data. We observe this when adjusting the time scale α for Example 1 of the main text. Starting with an α value of 0.5 leads to inaccurate learning and mismatched PSDs and transition time (Supplementary FIG. 9(a)). Decreasing α to 0.25 results in better predictions and similar PSDs (Supplementary FIG. 9(b)). Further decreasing α to 0.01 deteriorates model performance and PSD matching (Supplementary FIG. 9(c)). Thus, a better match of PSDs indicates better basin learning, allowing α adjustment without relying on prior basin information.

D. The robustness of the results under a range of hyperparameters

The hyperparameter α is the time scale in RC, and the β is the regularization parameter to avoid overfitting. These two hyperparameters are crucial for the present method. To assess the influence of changing the α and β in the present method, we adjust the α value around 0.2 and increase the β value from 1×10^{-8}, when keeping other hyperparameters constant.

Supplementary FIG. 18(a) demonstrates the trained slow-scale model under the values of $\alpha = 0.15$ and $\beta = 1 \times 10^{-8}$. The ten slow-scale time series correctly converge to their stable states, respectively, which means we can perform rolling prediction to predict noise-induced transitions. In the evaluation, we compare the transition time between 100 sets of test and the predicted data. The match between these two (Supplementary FIG. 19(a)) demonstrates that the present method is able to learn noise-induced transitions with $\alpha = 0.15$ and $\beta = 1 \times 10^{-8}$. As we adjust the value of α to 0.2 (Supplementary FIG. 18(b)) and 0.25 (Supplementary FIG. 18(c)), the obtained results are satisfactory (Supplementary FIG. 19(b, c)). Additionally, the convergence speed of the ten slowly time-scale series may have discrepancies compared with the actual dynamics, thus the results of the evaluation may contain biases.

The previous study [12] observed that β should not be too small, as an example, in the Fig. 2 of the mentioned study, the regularization parameter is 2.5×10^{-6}. Following this, we proceed to modify hyperparameter β by increasing it to 1×10^{-7}, and the results for α values of 0.15, 0.2 and 0.25 are depicted in Supplementary FIG. 18(d-f). These outcomes enable us to proceed to the predicting phase, and Supplementary FIG. 19(d-f) demonstrate that the transition time of predicted data matches the test data. As we increase the value of β to 1×10^{-6}, the results (Supplementary FIG. 18(g-i) and Supplementary FIG. 19(g-i)) also show the effectiveness of the present method.

E. Assessing the impact of training set length on performance

To assess the performance of the present method with the limited training data, we shorten the length of the training set based on the Example 1 of the main text. Additionally, we keep the length of the predicting set same as the training set. Supplementary FIG. 16(a, b) display the same results in FIG. 2(e, f) of the main text, over the same hyperparameters and length ($10000\delta t$). Next, we reduce the length of the training and predicting sets by half ($5000\delta t$), as illustrated in Supplementary FIG. 16(c, d). Without any significant changes in the results, we continue to reduce the length by half, this means that the training and predicting sets are only one-fourth of their original size ($2500\delta t$). However, the presented results (Supplementary FIG. 16(e, f)) exhibit larger errors compared with the results in Supplementary FIG. 16(a-d). Furthermore, we proceed to halve the length ($1250\delta t$) of both the training and predicting sets once again. The results depicted in Supplementary FIG. 16(g, h) demonstrate significant errors. This example shows the training set length impact on our training.

F. More Examples

1. A one-dimensional tilted bistable gradient system

To demonstrate whether the noise-induced transitions can be predicted by the present method for systems with the tilted double-well potential, we apply the present method to the system depicted in Supplementary FIG. 10. This system is the same as the Example 1 of the main text, and the parameter $c = 0.25$ makes the system tilted. The distinct time scales of upward and downward transitions necessitate two sets of hyperparameters for effective learning. The "upward transitions" refers to the transitions from down state to up state, and the "downward transitions" refers to the transitions from up state to down state.

Supplementary FIG. 10(a) shows that we focus on the upward transitions, and Supplementary FIG. 10(b-f) are the results. As the same method in Framework, we find a proper hyperparameters set 1 (for upward transitions) listed in Supplementary Table IV to obtain the trained slow-scale model (for upward transitions). The ten different slowly time-scale series and separated noise distribution are illustrated in Supplementary FIG. 10(b). In the predicting phase, we perform rolling prediction with the trained slow-scale model (for upward transitions) and the noise distribution. Then, we evaluate the transitions within the test and the predicted data.

We note a significant difference in the downward transition time between test and predicted data (Supplementary FIG. 10(c)), and with differences in the number of downward transitions in $10000\delta t$ (Supplementary FIG. 10(d)). Meanwhile, our evaluation of the upward transition time (Supplementary FIG. 10(e)) and the number of upward transitions in $10000\delta t$ (Supplementary FIG. 10(f)) match. It indicates the effectiveness of hyperparameters set 1 for upward transitions.

Using the same training set, we switch to downward transitions, with results shown in Supplementary FIG. 10(g-l). As we shift focus, we observe the results similar to the upward case. With the proper hyperparameters set 2 (in Supplementary Table IV) for downward transitions, we determine the corresponding slow-scale model (for downward transitions). The results, including downward transition time (Supplementary FIG. 10(i)) and the number of downward transitions in $10000\delta t$ (Supplementary FIG. 10(j)) match. Conversely, for upward transitions, a distinct error is observable in Supplementary FIG. 10(k), while Supplementary FIG. 10(l) shows the result with an insignificant error. It displays the effectiveness of hyperparameters set 2 for downward transitions.

2. A two-dimensional bistable gradient system

Before the work in the Example 3 of the main text, we design a pre-experiment, to ensure the present method can apply in a 2D bistable system. The system has noise-induced transitions between the two potential wells under the noise as illustrated in Supplementary FIG. 11(a), and the scatter plot of the training set (consisting of 20000 data points) is depicted in Supplementary FIG. 11(b). According to the method in Framework, we generate ten different slowly time-scale series in the training phase (Supplementary FIG. 11(c)) and prediction (consisting of 20000 data points) in the predicting phase (Supplementary FIG. 11(d)). In Supplementary FIG. 11(e, f), we compare the number of transitions and the transition time (over $20000\delta t$) of generated data and predictions. The proper hyperparameters for this system are listed in Supplementary Table V.

3. A two-dimensional tilted bistable gradient system

To demonstrate that the present method can predict noise-induced transitions in a 2D tilted system, we first apply it to a 2D bistable gradient system with white noise as illustrated in Supplementary FIG. 12.

Similar to the 1D tilted double-well potential system, the upward and downward transitions necessitate two sets of hyperparameters. As shown in Supplementary FIG. 12, the same graphical scheme used in Supplementary FIG. 10 is applied to represent the results of the two-dimensional tilted bistable gradient system with Gaussian white noise. Supplementary FIG. 12(a-f) show the results for upward transitions, and the hyperparameters set 1 (for upward transitions) are listed in Supplementary Table VI. Supplementary FIG. 12(g-l) show the results for downward transitions, and the hyperparameters set 2 (for downward transitions) are listed in Supplementary Table VI.

4. A two-dimensional tilted bistable non-gradient system

To demonstrate that the present method can predict noise-induced transitions in a both tilted and non-gradient system, we add a rotation element to make this system without detailed balance as illustrated in Supplementary

FIG. 13 to apply our approach.

As shown in Supplementary FIG. 13, the same graphical scheme used in Supplementary FIG. 12 is applied to represent the results of a two-dimensional non-gradient system with double-well potential and Gaussian white noise. Supplementary FIG. 13(a-f) show the results for upward transitions, and the hyperparameters set 3 (for upward transitions) are listed in Supplementary Table VI. Supplementary FIG. 13(g-l) show the results for downward transitions, and the hyperparameters set 4 (for downward transitions) are listed in Supplementary Table VI.

5. A two-dimensional tristable system

To assess the performance of the present method in multi-stable systems, we study a 2D tristable system [13] as illustrated in Supplementary FIG. 14(a):

$$\begin{aligned}\dot{u}_1 &= 10u_1 e^{-u_1^2}\left[e^{-(u_2-1/3)^2} - e^{-(u_2-5/3)^2}\right] - 8e^{-u_2^2}\left[(u_1-1)e^{-(u_1-1)^2} + (u_1+1)e^{-(u_1+1)^2}\right] \\ &\quad - 0.8u_1^3 + \sqrt{\frac{2}{\gamma}}\xi_1(t), \ \ t \geq 0,\end{aligned} \tag{S1}$$

$$\begin{aligned}\dot{u}_2 &= -8u_2 e^{-u_2^2}\left[e^{-(u_1-1)^2} + e^{-(u_1+1)^2}\right] + 10e^{-u_1^2}\left[(u_2-\tfrac{1}{3})e^{-(u_2-1/3)^2} - (u_2-\tfrac{5}{3})e^{-(u_2-5/3)^2}\right] \\ &\quad - 0.8(u_2-\tfrac{1}{3})^3 + \sqrt{\frac{2}{\gamma}}\xi_2(t), \ \ t \geq 0.\end{aligned} \tag{S2}$$

The system has noise-induced transitions under the Gaussian white noise $(\xi_1(t), \xi_2(t))$, and γ corresponds to the noise strength.

To investigate transitions among three stable states, we specifically consider the u_1 direction due to the overlapping of two wells in the u_2 direction. Therefore, our study focuses on a one-dimensional time series in the u_1 direction. We generated a time series from Eqs. (S1) and (S2), in $t \in [0, 100]$. The time series is plotted for the first $30000\delta t$ in Supplementary FIG. 14(b).

In the training phase, with the training set $t \in [0, 50]$, we obtain the appropriate hyperparameters listed in Supplementary Table VII. The time series from ten distinct initial points converge to the stable states ($u_1 = -1, 0, 1$) as illustrated in Supplementary FIG. 14(c). Subsequently, we perform rolling prediction for $t \in [50, 80]$ using the separated noise and the trained slow-scale model. The result demonstrates that the method learns the multi-state dynamics of the generated time series, including the transitions among these stable states.

6. A bistable gradient system with high-dimensional colored noise

To demonstrate that the present method can learn a single stochastic transition under high-dimensional colored noise, we consider a system Eqs. (S3) to (S7) studied in [14]:

$$\dot{u}_1 = u_1(1-u_1^2) + B\cos(\Omega t) + \sigma(\mu_1 - \langle \mu_1 \rangle), \tag{S3}$$

$$\dot{\mu_1} = \frac{1}{\epsilon}((\mu_2 - \mu_{K-1})\mu_K) - \mu_1 + F, \tag{S4}$$

$$\dot{\mu_2} = \frac{1}{\epsilon^2}((\mu_3 - \mu_K)\mu_1 - \mu_2 + F), \tag{S5}$$

$$\dot{\mu_i} = \frac{1}{\epsilon^2}((\mu_{i+1} - \mu_{i-2})\mu_{i-1} - \mu_i + F), \ \ i = 3, \ldots, K-1, \tag{S6}$$

$$\dot{\mu_K} = \frac{1}{\epsilon^2}((\mu_1 - \mu_{K-2})\mu_{K-1} - \mu_K + F). \tag{S7}$$

The parameters B, ϵ and σ correspond to the strength of the noise, while Ω controls the frequency of the cosine term. The $K = 36$ and $F = 8$ lead the Lorenz-96 system $(\mu_1, \ldots, \mu_K)$ to chaotic dynamics. The $\langle \mu_1 \rangle$ means the average of μ_1, and $\mu_1 - \langle \mu_1 \rangle$ makes the mean of the noise influenced u_1 to become zero. We generated a time series of length $16000\delta t$. The training set is $t \in [0, 80]$, as illustrated in Supplementary FIG. 15(a), where a noise-induced transition occurs in $t \in [19.8, 24.3]$, and the predicting set is $t \in [80, 160]$.

In the training phase, we obtain ten distinct slowly time-scale series converged to their corresponding state (Supplementary FIG. 15(b)), and we separate noisy series. The hyperparameters are listed in Supplementary Table VIII Set 1. In the predicting phase, we utilize a second reservoir (in FIG. 3(a) of the main text) to learn the noisy data

separated in the training phase, and the proper hyperparameters are listed in Supplementary Table VIII Set 2. With the slow-scale model and learned noisy series, we execute a rolling prediction to predict transition.

In the evaluation, we replicate 50 times with the same hyperparameters. These predictions are then used alongside trained slow-scale model for 50 times rolling prediction. The average of the predictions approximates the test data (Supplementary FIG. 15(c)). Furthermore, Supplementary FIG. 15(d) shows the average absolute error between the 50 predictions and the actual time series. The result demonstrates that the method can nearly capture the stochastic transition with high-dimensional noise.

II. SUPPLEMENTARY FIGURES

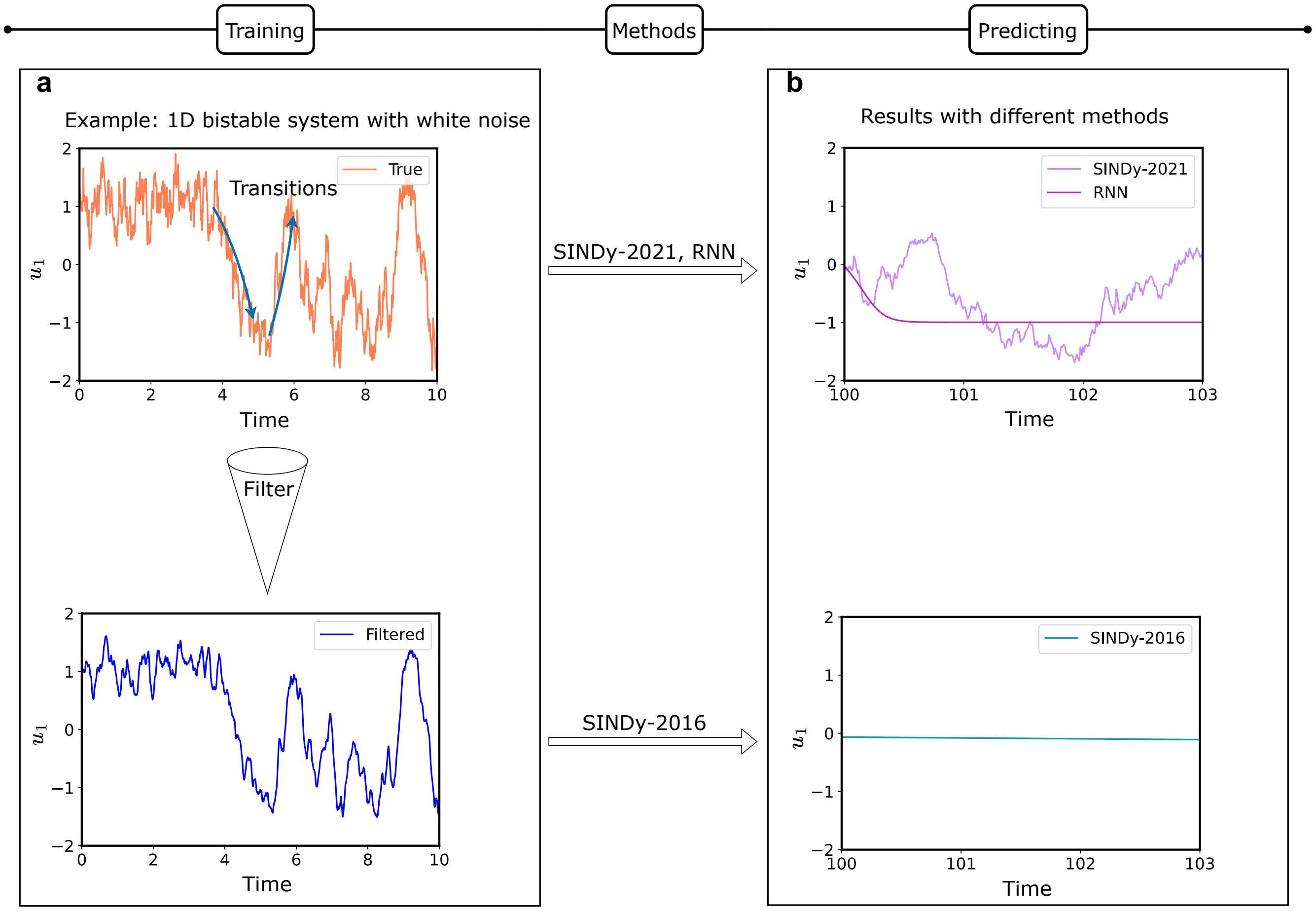

Supplementary FIG. 1. **The conventional approaches do not capture stochastic transitions.** (a) Training data for these methods. The length of the training set is 100, the same as in the the Example 1 of the main text. Upper: a piece of generated data from Eq. (9) of the main text as the training set of SINDy-2021 and RNN to learn noise-induced transitions. Lower: filtered data from upper for SINDy-2016 to learn. (b) Results of the predictions. Upper: predicted data from SINDy-2021 (pale purple) and RNN (deep purple), these methods do not learn noise-induced transitions. Lower: the identification by SINDy-2016 (cyan), SINDy-2016 does not predict the filtered data. More details are in Supplementary FIG. 2, Supplementary FIG. 3 and Supplementary FIG. 4.

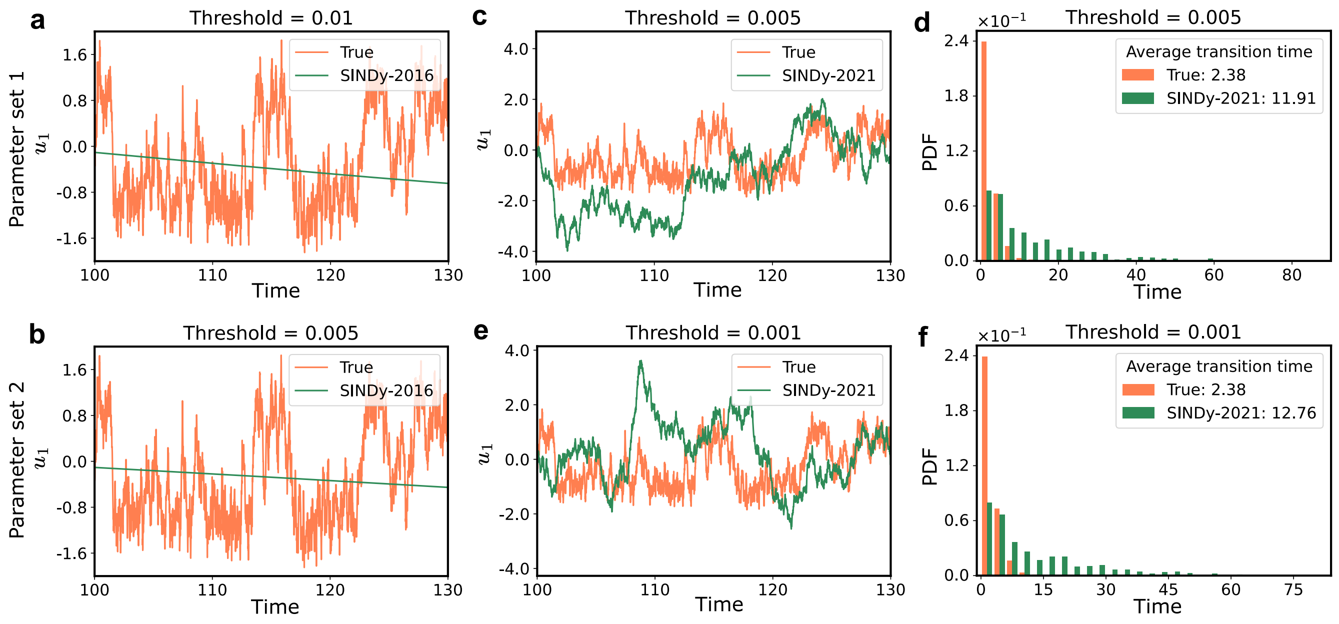

Supplementary FIG. 2. **Learning stochastic transitions by SINDy.** The training set is generated from Eq. (9) of the main text (FIG. 2(b) of the main text). The hyperparameters: $\delta t = 0.01$, Order = 3, Library function = Polynomial function. (a) The result of SINDy-2016 [1, 2] (threshold = 0.01) to data with noise, and generation by the function SINDy identified (green line) compares with actual data (coral line). The prediction does not match the true data. (b) Result same as (a), and threshold = 0.005. (c) The result of applying SINDy-2021 [3] (threshold = 0.005) to identify the dynamics and separate noise distribution. Prediction (green line) generated by the identified function and noise sampled from the distribution compares with the actual time series (coral line). (d) Histograms of transition time for actual and predicted data of (c). The transition time of the predictions significantly exceeds the actual series. (e, f) Results same as (c, d), and threshold = 0.001.

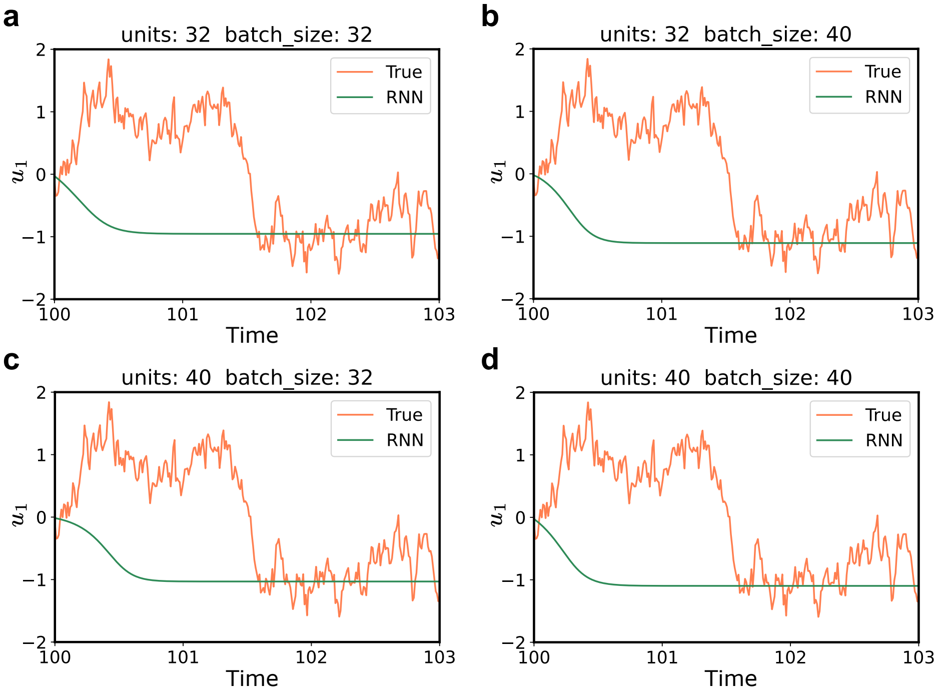

Supplementary FIG. 3. **Utilizing RNN to predict noise-induced transitions.** The training set is the same as the the Example 1 of the main text. The hyperparameters for RNN (LSTM) are: epoch = 10, units and batch size are adjusted. (a) The true (coral line) and predicted data (green line) from trained LSTM (units = 32, batch size = 32) are illustrated, $t \in [100, 103]$. (b) The actual data and the prediction with units = 32, batch size = 40. (c) The actual data and the prediction with units = 40, batch size = 32. (d) The actual data and the prediction with units = 40, batch size = 40. These four outcomes present significant differences from the true data.

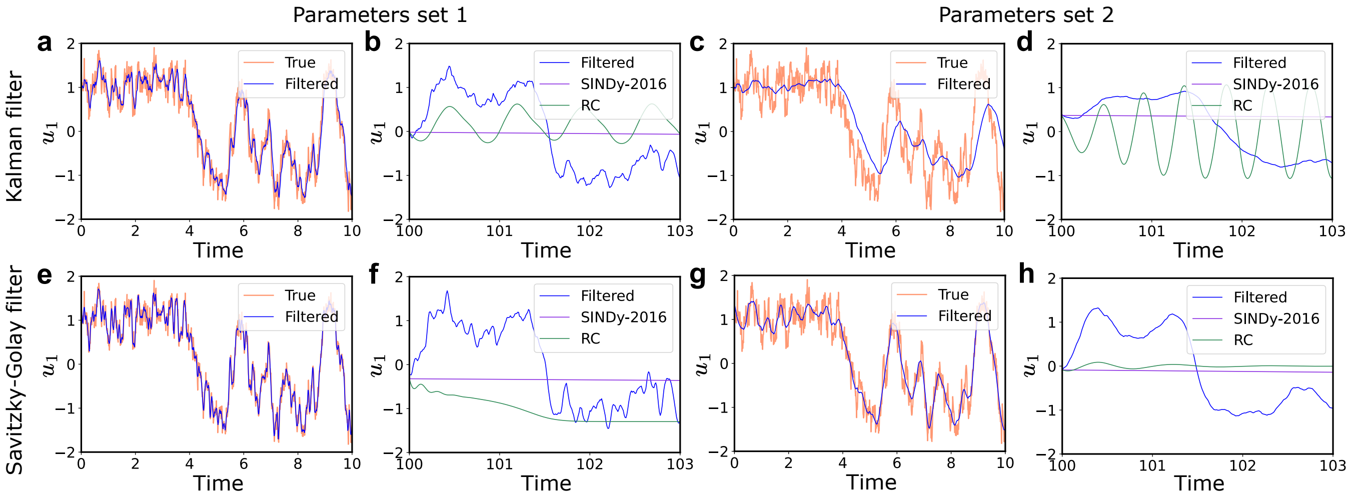

Supplementary FIG. 4. **Predicting time series preprocessed by filters.** The data with noise is generated from Eq. (9) of the main text. (a) The actual time series (coral line) and the filtered data (blue line) preprocessed by the Kalman filter (measurement noise covariance matrix $Q = 0.05$). The filtered series is the training data of SINDy-2016 and RC. (b) Results of the predicted data from SINDy-2016 (purple line) and RC (green line) compared to the filtered series. The prediction is significantly different from the filtered data. (c, d) Results are the same as (a, b), and $Q = 0.0005$. (e) Result of preprocessing the time series by the Savizky-Golay filter (window length $W = 10$, polynomial order $P = 2$). (f) Results of the test and predicted data (SINDy-2016 and RC) also exhibit differences. (g, h) Results same as (e, f), $W = 50$ and $P = 2$.

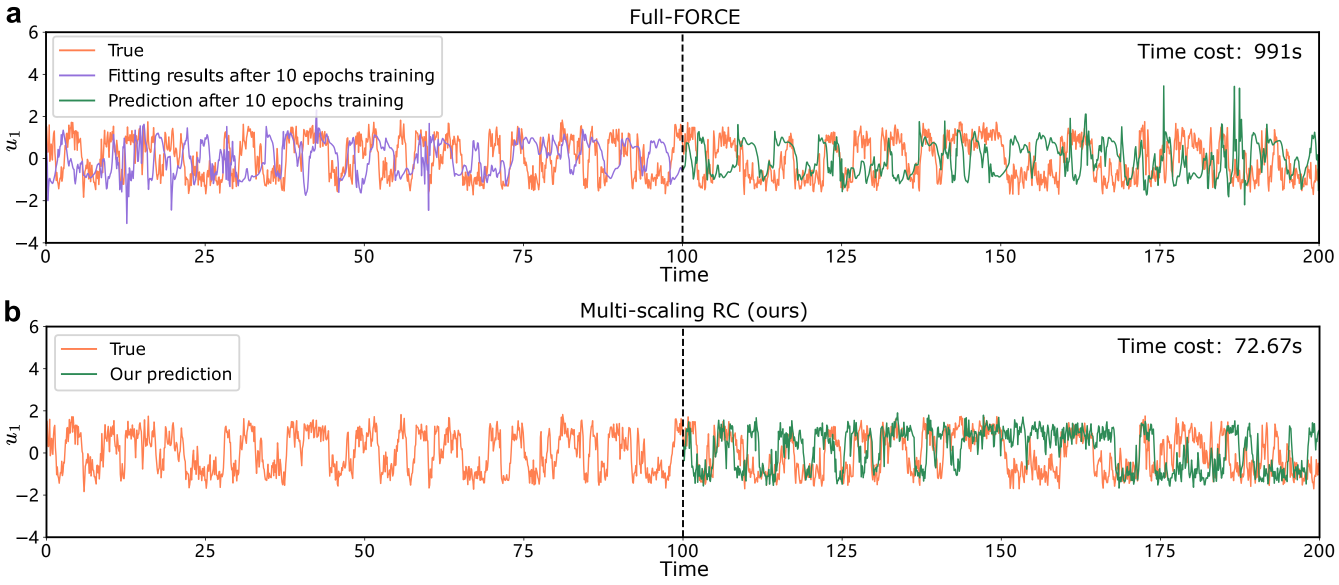

Supplementary FIG. 5. **FORCE learning (full-FORCE model) and present method for a one-dimensional bistable system.** The system is Example 1 of the main text. The training data (before the dark dashed line) is $t \in [0, 100]$, with data points plotted every 10 time steps. (a) Prediction in $t \in [100, 200]$ of the full-FORCE model [9], with the same hyperparameters as in Figure 4 of [9]. The predicted data show larger fluctuations compared to the true data. (b) Our prediction has similar fluctuations to the true data, requiring less time than the full-FORCE model.

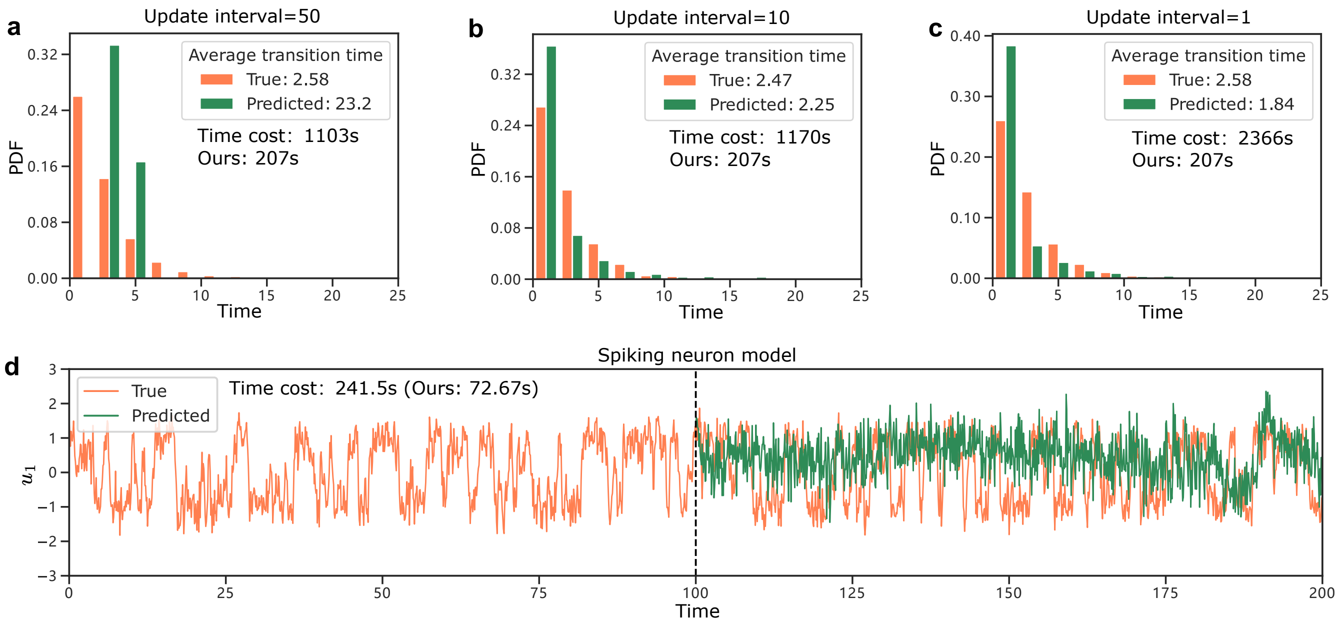

Supplementary FIG. 6. **FORCE learning (spiking neuron model) for a one-dimensional bistable system with various update intervals.** Update interval: FORCE updates are applied every update interval time steps. The system is the Eq. (9) of the main text. Training set: $t \in [0, 100]$, predicting set: $t \in [100, 2000]$. The hyperparameters are the same as in Figure 6 of [9]. Histograms of transition time for test and predicted data with update intervals of (a) 50, (b) 10, and (c) 1. (d) Prediction in $t \in [100, 200]$ of using spiking neuron model [9] (after the black dashed line), update interval = 10. Data points are plotted every 10 time steps.

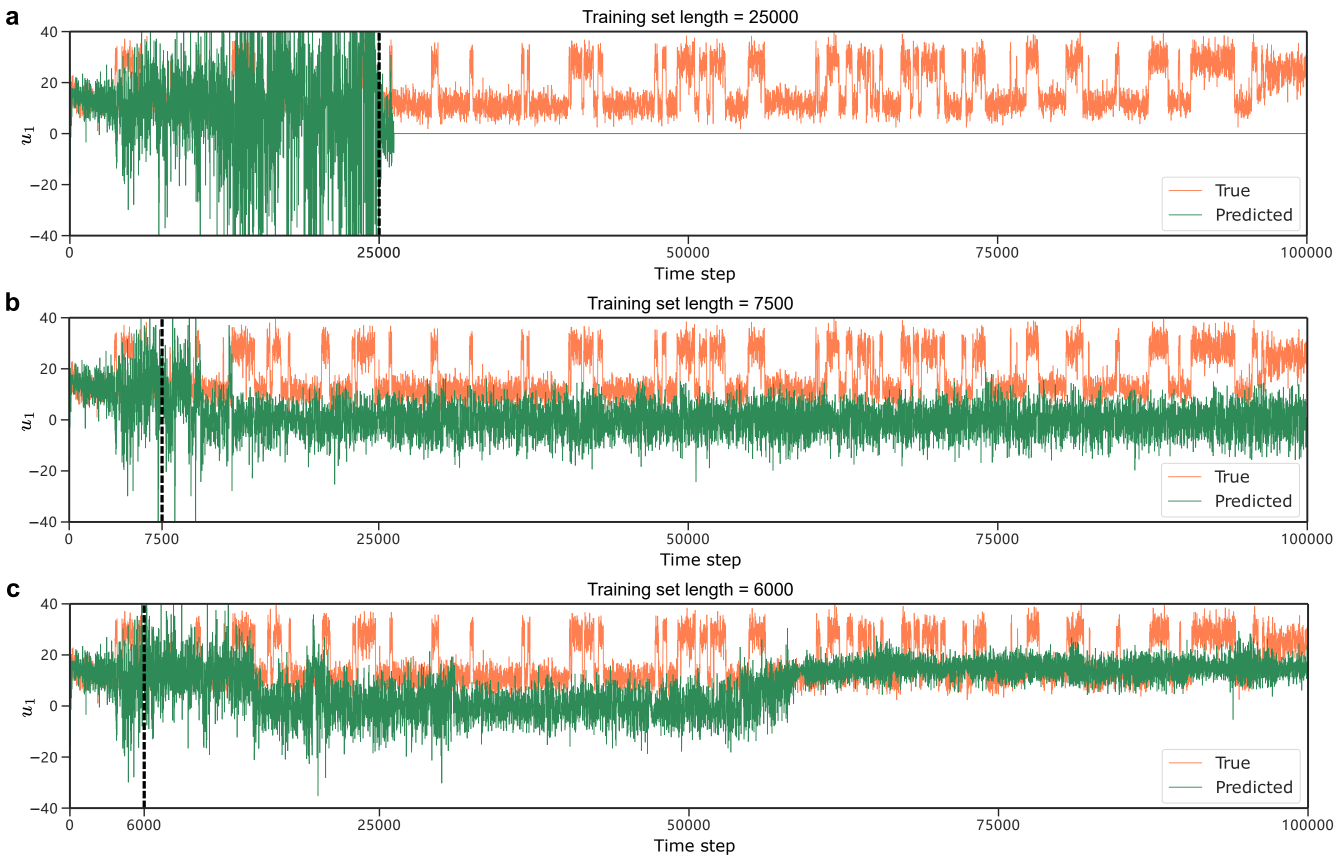

Supplementary FIG. 7. **FORCE learning (spiking neuron model) for the example of protein folding by using various lengths of training data.** The training data is same as the Example 4 of the main text, update interval = 10, and other hyperparameters are the same as those in Supplementary FIG. 6(b). Black dashed lines represent the boundary between the training and predicting sets. (a) Training set length (T_{train}): 25000 time steps; predicted data converges to zero. (b) T_{train}: 7500 time steps; predicted data show no stochastic transitions. (c) T_{train}: 6000 time steps; predicted data show no stochastic transitions.

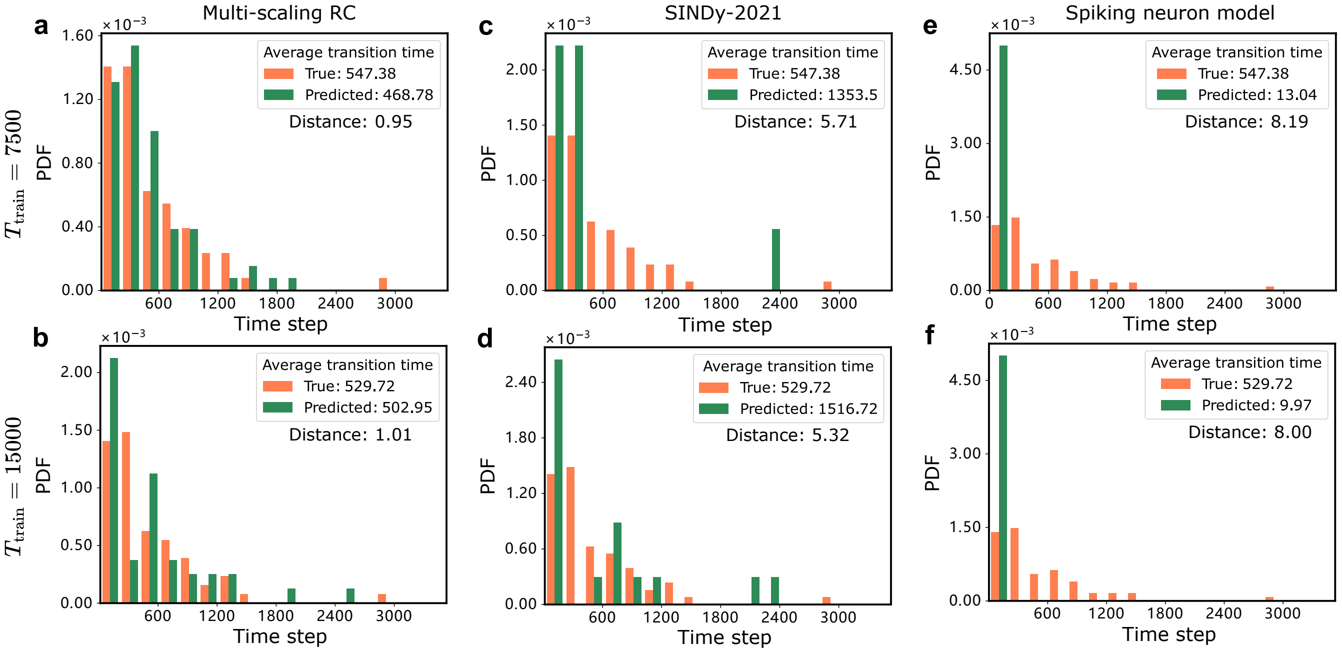

Supplementary FIG. 8. **Histograms of transition time (downward) for different methods on Example 4 of the main text.** Training set lengths (T_{train}) are (a, c, e) 7500 and (b, d, f) 15000 time steps, and prediction length is 100000 time steps. Distance: symmetric Kullback-Leibler (KL) divergence. (a, b) Multi-scaling RC: predictions match true data with small distance values. (c, d) SINDy-2021 (Threshold = 0.001, Order = 4) [2]: larger distance values and prediction errors. (e, f) FORCE learning: fails to capture transition time accurately, showing large distance values. The hyperparameters are same in Supplementary FIG. 7.

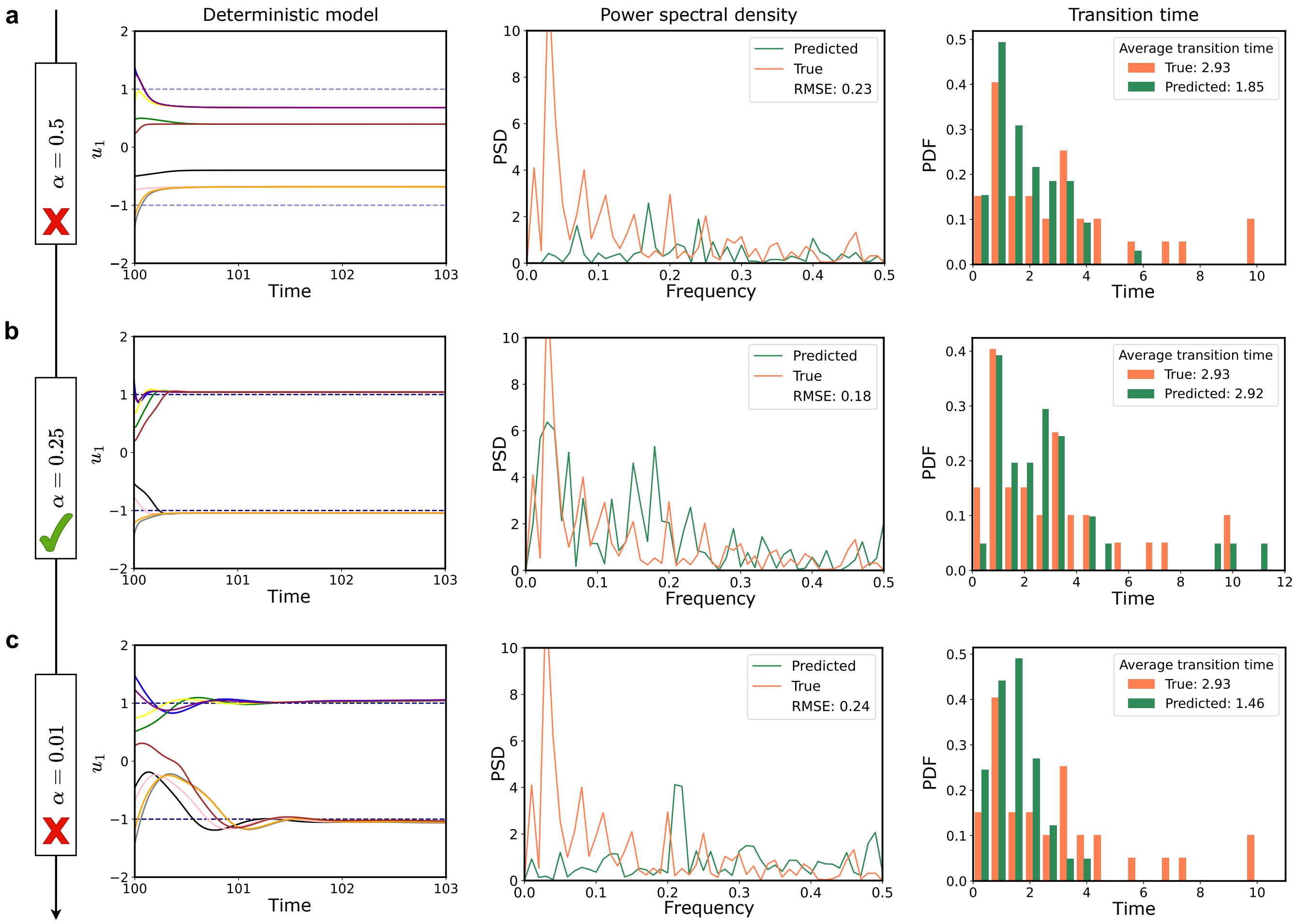

Supplementary FIG. 9. **Power spectral density (PSD) analysis can be an indicator for accurate training.** The system is Example 1 of the main text. Discrepancies of PSD are quantified using the root mean square error (RMSE). Green checkmark: good performance. Red cross: poor performance. (a) The trained slow-scale model, when $\alpha = 0.5$. The PSDs of the training data (coral line) and the predicted data (green line) exhibit a poor match, with RMSE 0.23. The transition time has large deviations. (b) The trained slow-scale model when $\alpha = 0.25$. The PSD of the training data matches that of the predicted data, with RMSE 0.18. The transition time of the prediction closely matches the training data. (c) Results when $\alpha = 0.01$, neither the transition time nor the PSD of the predicted data align with those of the training data, suggesting that $\alpha = 0.01$ is not optimal.

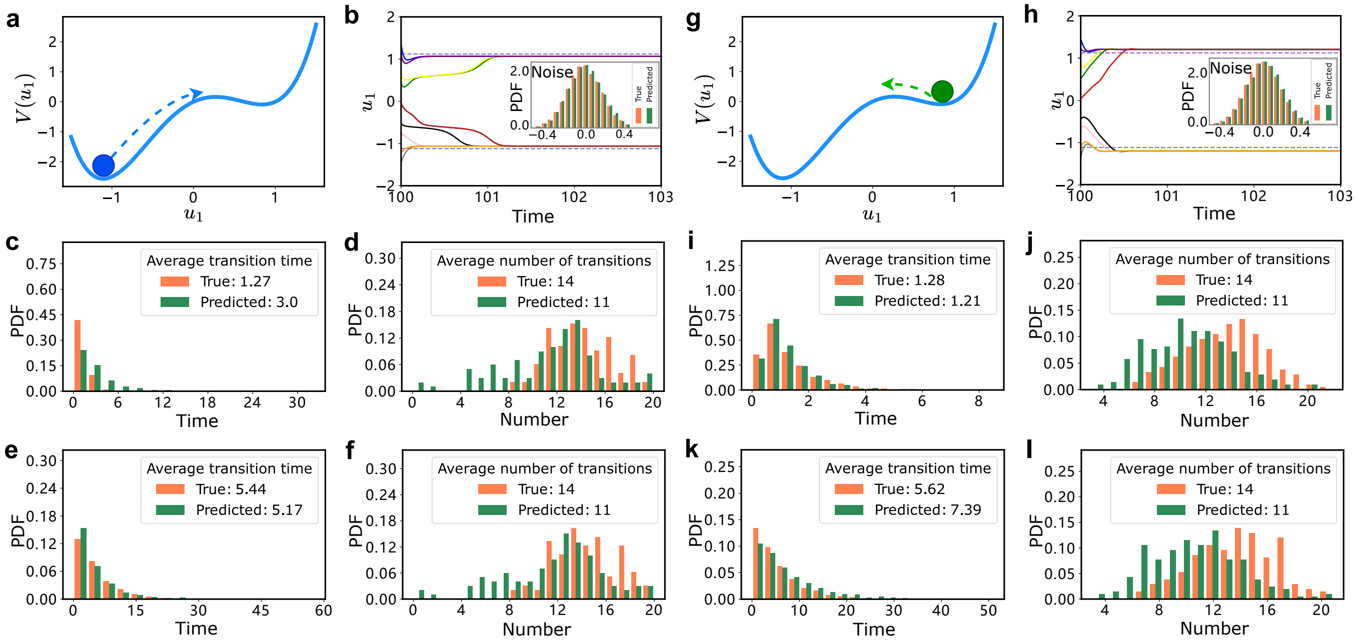

Supplementary FIG. 10. **Capturing stochastic transitions in a 1D tilted bistable gradient system with white noise.** The system is the same as the the Example 1 of the main text, and the parameter $c = 0.25$ makes the potential tilted. Generated time series from Eq. (9) of the main text ($b = 5, c = 0.25, \varepsilon = 0.3, \delta t = 0.01$), spanning a duration of $20000\delta t$, with the training set $t \in [0, 100]$ and the predicting set $t \in [100, 200]$. (a) Schematic of noise-induced upward transitions in the 1D tilted bistable gradient system with Gaussian white noise. For figures (b-f), we focus on the upward transitions. (b) The trained slow-scale model transforms ten different start points into ten different slowly time-scale series (color lines), and the noise distribution is separated in the training phase. (c) Histograms of downward transition time for the test and predicted data. (d) The number of downward transitions for the test and predicted data. The duration of the predicting set is $10000\delta t$ (10000 data points). (e) Histograms of upward transition time for the test and predicted data. (f) The number of upward transitions for the test and predicted data. (g-l) Results focusing on the downward transitions.

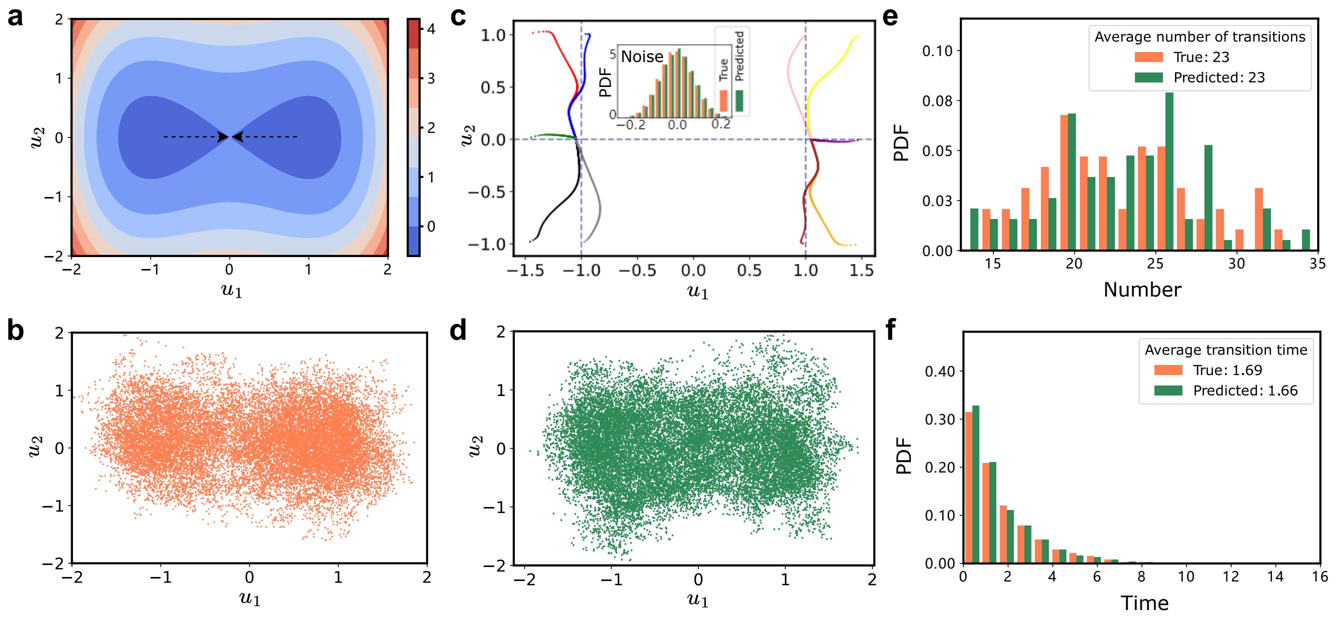

Supplementary FIG. 11. **Capturing stochastic transitions in a 2D bistable gradient system with white noise.** The system is the same as the Example 3 of the main text, and the parameter $a = 0$. (a) Schematic of noise-induced transitions in the 2D bistable gradient system with Gaussian white noise. (b) Generated time series from Eqs. (14) and (15) of the main text ($a = 0, b = 5, c = 0, \varepsilon_1 = \varepsilon_2 = 0.3, \delta t = 0.002$) with $t = 80$ as ground truth. (c) The trained slow-scale model transforms ten different start points into ten different slowly time-scale series (color lines), $t \in [40, 80]$, and the noise distribution is separated in the training phase. (d) Result of prediction in $t \in [40, 80]$ using the slow-scale model and noise in (c). (e) The number of transitions for the 100 replicates simulated in $t \in [40, 80]$ and the 100 generated matches. The transition refers to the time series in the u_1-direction that crosses the zero point and without returning for $50\delta t$. (f) Histograms of transition time for the test and predicted data. When a transition occurs within the system, the transition time is defined as the interval between two consecutive zero crossings in the u_1 direction.

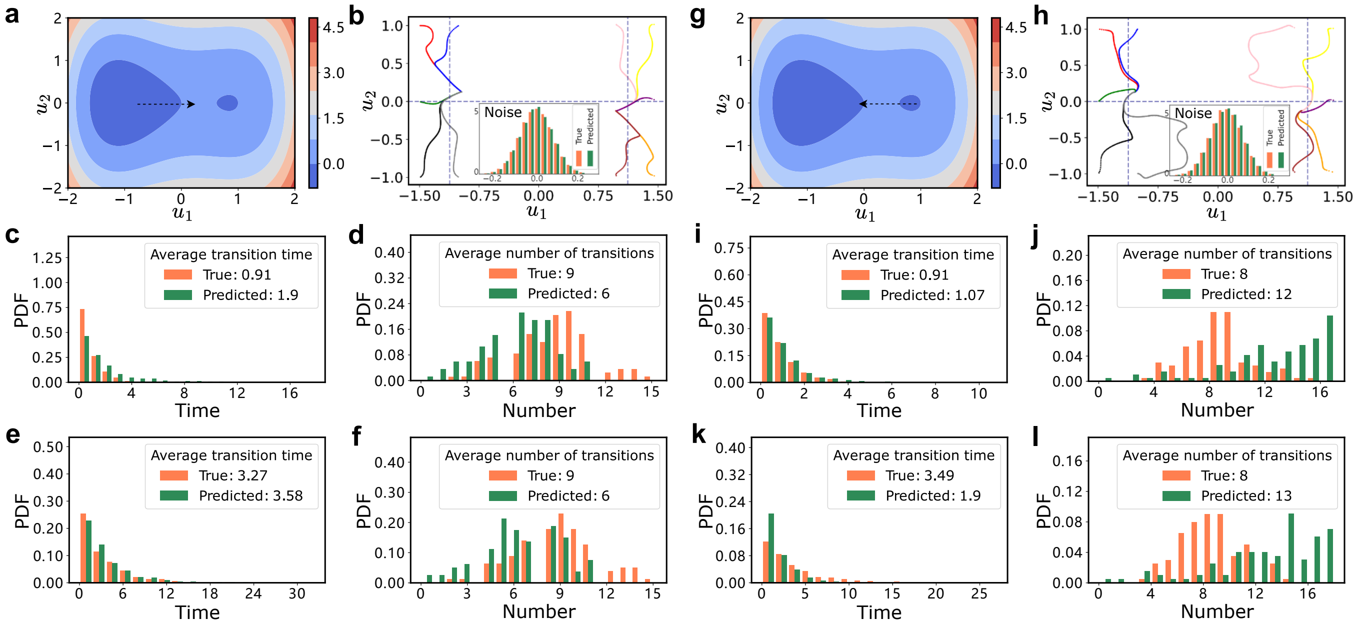

Supplementary FIG. 12. **Capturing stochastic transitions in a 2D tilted bistable gradient system with white noise.** The system is the same as the Example 3 of the main text, with the parameter $c = 0.25$ making the potential tilted, $a = 0$, and $\delta t = 0.002$. (a) Schematic of noise-induced upward transitions in the 2D tilted bistable gradient system. For figures (b-f), we focus on the upward transitions. (b) The trained slow-scale model transforms ten different start points into ten different slowly time-scale series (color lines), $t \in [40, 80]$, and the noise distribution is separated in the training phase. (c) Histograms of downward transition time for the test and predicted data. (d) The number of downward transitions for the test and predicted data. The duration of the predicting set is $20000\delta t$ (20000 data points). (e) Histograms of upward transition time for the test and predicted data. (f) The number of upward transitions for the test and predicted data. (g-l) Results focusing on the downward transitions.

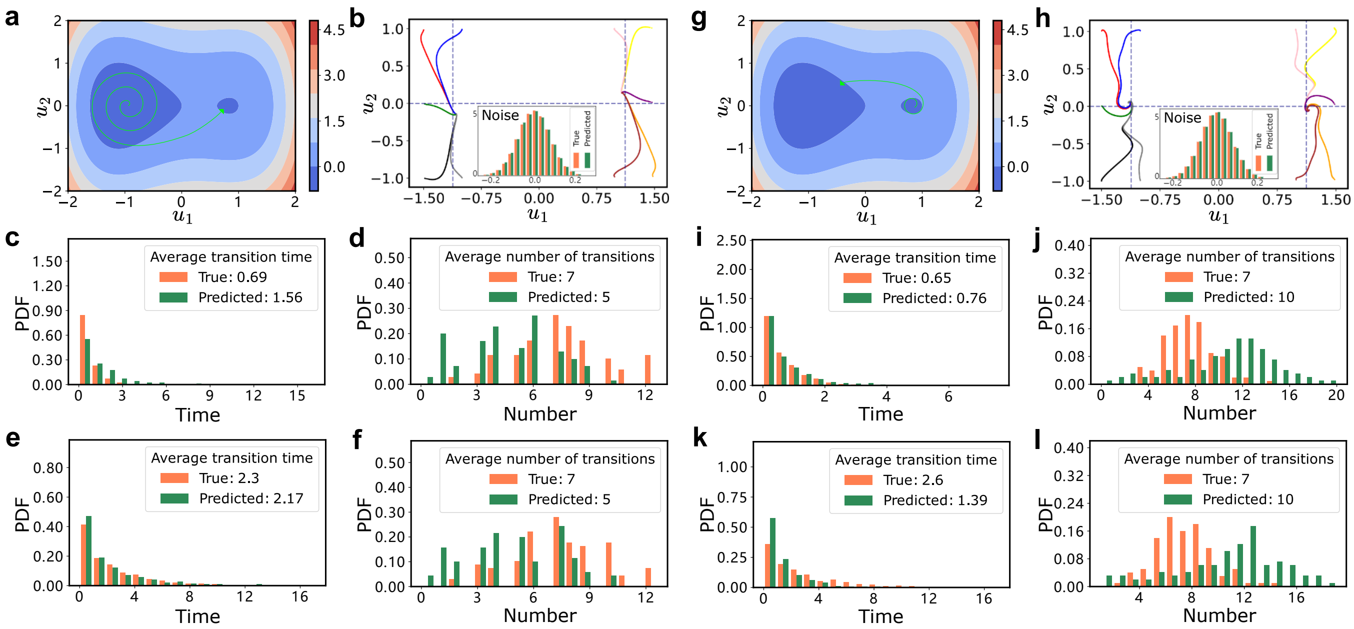

Supplementary FIG. 13. **Learning noise-induced transitions in a 2D tilted bistable non-gradient system.** The system is the same as the Example 3 of the main text, and the parameter $c = 0.25$ makes the potential tilted. The $a = 2$ represents the strength of the non-detailed balance part, $\delta t = 0.001$. (a) Schematic of noise-induced upward transitions in the 2D tilted bistable non-gradient system with Gaussian white noise. For figures (b-f), we focus on the upward transitions. (b) The trained slow-scale model transforms ten different start points into ten different slowly time-scale series (color lines), $t \in [25, 50]$, and the noise distribution is separated in the training phase. (c) Histograms of downward transition time for the test and predicted data. (d) The number of downward transitions for the test and predicted data. The duration of the predicting set is $25000\delta t$ (25000 data points). (e) Histograms of upward transition time for the test and predicted data. (f) The number of upward transitions for the test and predicted data. (g-l) Results focusing on the downward transitions.

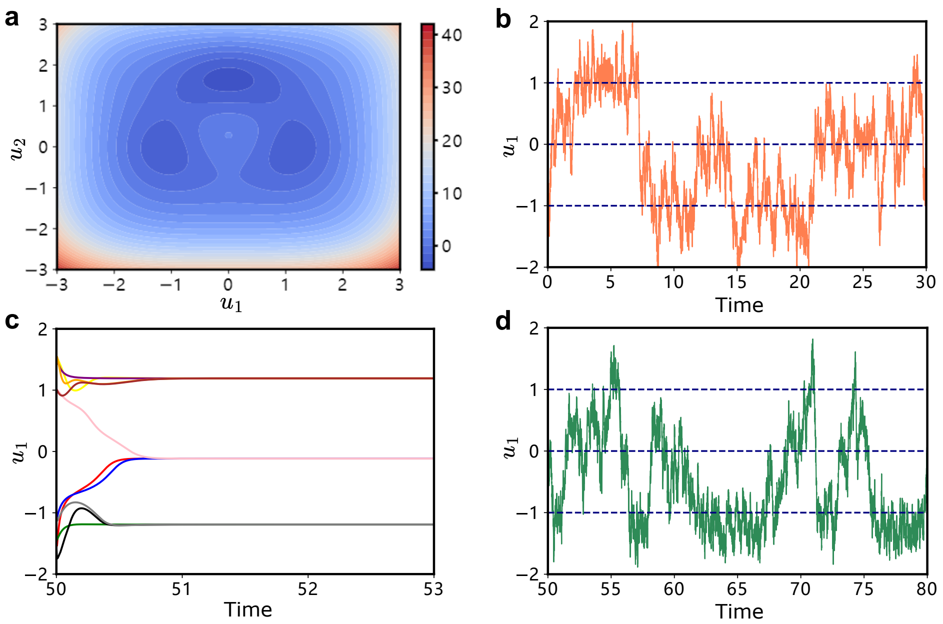

Supplementary FIG. 14. **Capturing stochastic transitions in a 2D tristable gradient system with white noise.** (a) Schematic of the 2D tristable system. (b) Generated time series from the Eqs. (S1) and (S2) ($\gamma = 1, \delta t = 0.001$) of [13] with $t = 30$ as ground truth. In the scenario where $\gamma = 1$, the three potential wells exhibit comparable depths. (c) The trained slow-scale model transforms ten different start points into ten different slowly time-scale series (color lines), only the u_1 direction is depicted. (d) Result of prediction using the slow-scale model and separated noise in $t \in [50, 80]$.

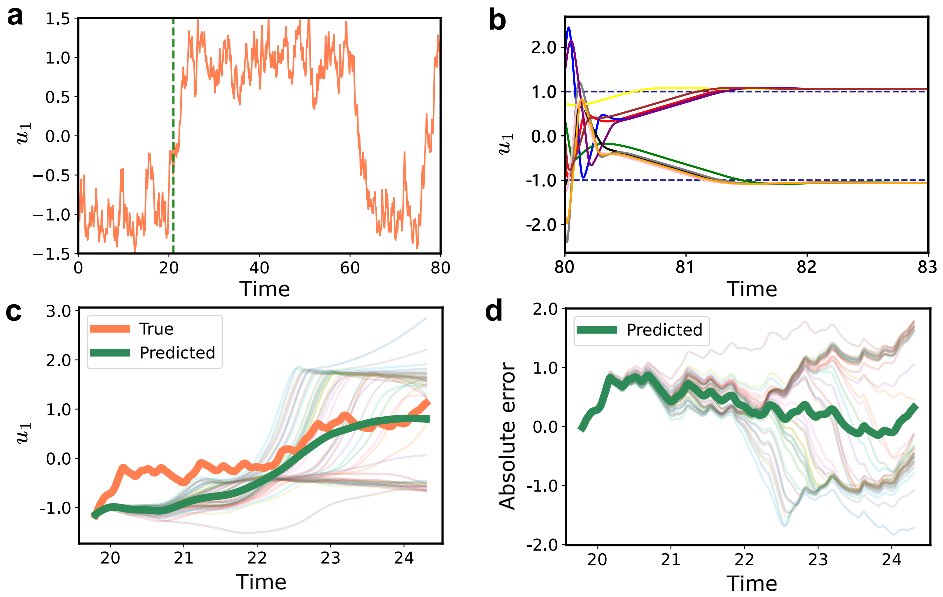

Supplementary FIG. 15. **Learning accurate transition time series in a bistable system with high-dimensional colored noise.** (a) Generated time series from the Eqs. (S3) to (S7), $(u_1(0) = -1, F = 8, K = 36, \mu_i(0) = F, 1 \leq i \leq K$ except $i = 20, \mu_{20}(0) = F + 0.01, B = 0.01, \Omega = 12, \epsilon = 0.35, \sigma = 0.6, \delta t = 0.01)$ with $t = 8000\delta t$ as ground truth, where a noise-induced transition occurs in $t \in [19.8, 24.3]$ marked by green dashed line. The noise data from $1000\delta t$ before the stochastic transition at $t = 19.8$ is applied to predict the noisy time series in $t \in [19.8, 24.3]$. (b) The trained slow-scale model transforms ten different start points into ten different slowly time-scale series (color lines), and separates noisy series in the training phase. (c) By repeating the process in FIG. 3(a) of the main text 50 times with the same hyperparameters, 50 predictions are illustrated (fainter lines). The average predicted time series (thick green) matches the test data. (d) The absolute error of the 50 predictions and their mean value (thick green).

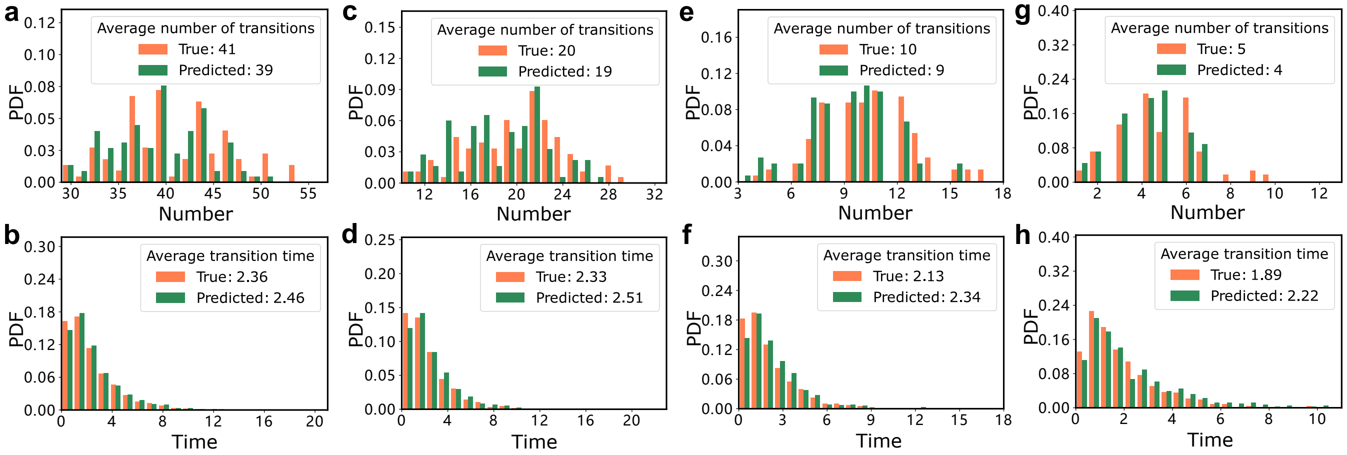

Supplementary FIG. 16. **The influence of decreasing the length of training and predicting sets based on the the Example 1 of the main text.** PDF: probability density function. (a) The number of transitions over the 10000 test and predicted data points. (b) Histograms of transition time of the 10000 test and predicted data points. (c, d) Results over the 5000 test and predicted data points. (e, f) Results over the 2500 test and predicted data points. (g, h) Results over the 1250 test and predicted data points.

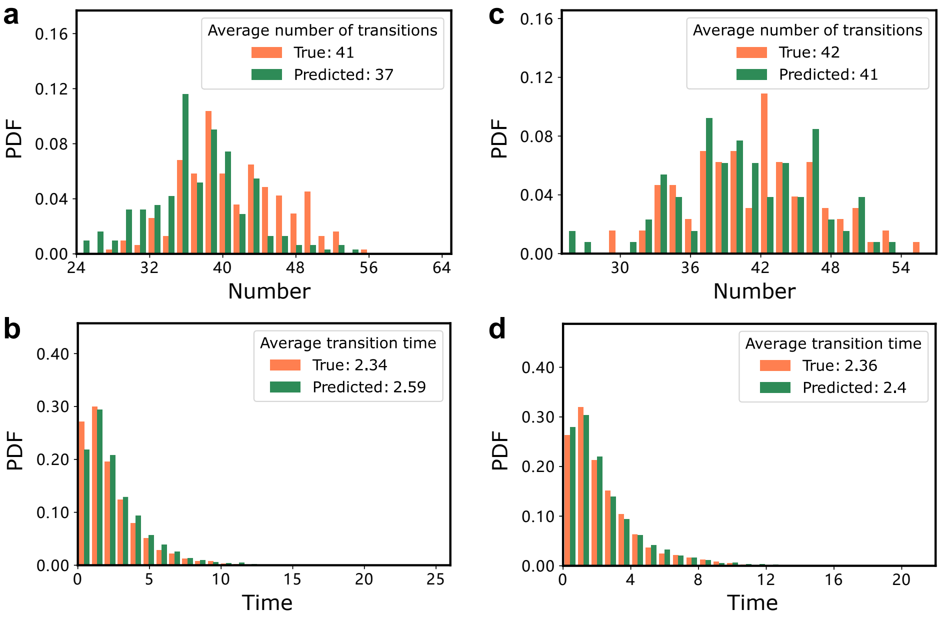

Supplementary FIG. 17. **Results of increasing the intensity of sampled noise in rolling prediction based on the the Example 1 of the main text.** PDF: probability density function. (a) The number of transitions of test and predicted data over $10000\delta t$. (b) Histograms of the transition time of the test and predicted data within $10000\delta t$. (c, d) The results with the sampled noise amplified by a factor of 1.1 in rolling prediction.

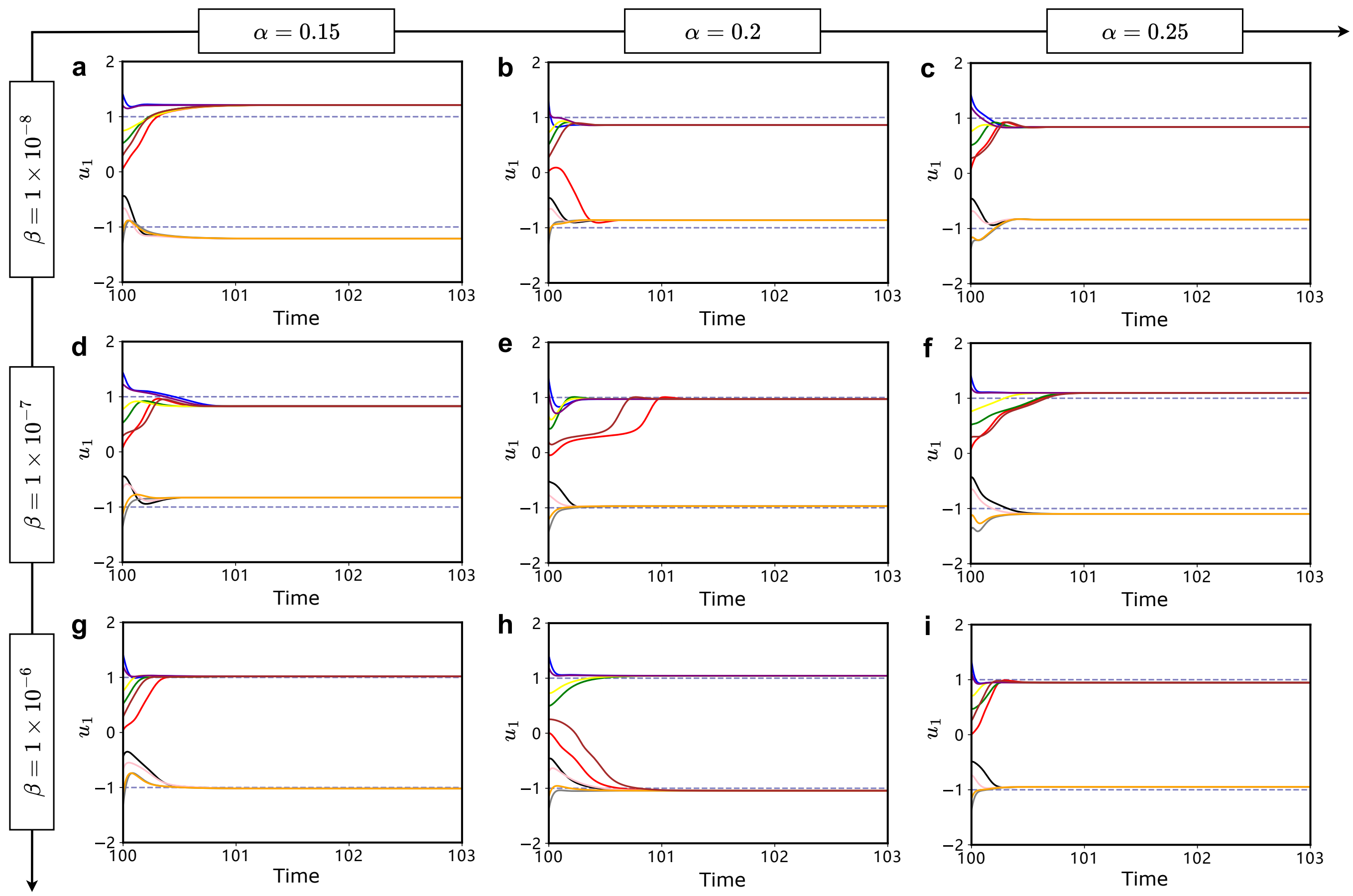

Supplementary FIG. 18. **The robustness of the results in the training phase under the hyperparameters α and β of the RC.** In the Example 1 of the main text, the hyperparameters $\alpha = 0.2, \beta = 1 \times 10^{-8}$. (a) The trained slow-scale model transforms ten different initial points into ten different slowly time-scale series with $\alpha = 0.15, \beta = 1 \times 10^{-8}$. It presents a favorable outcome. (b) Result with $\alpha = 0.2, \beta = 1 \times 10^{-8}$. (c) Result with $\alpha = 0.25, \beta = 1 \times 10^{-8}$. (d-f) The results are the same as in (a-c), with $\beta = 1 \times 10^{-7}$. (g-i) Results same as (a-c), while $\beta = 1 \times 10^{-6}$.

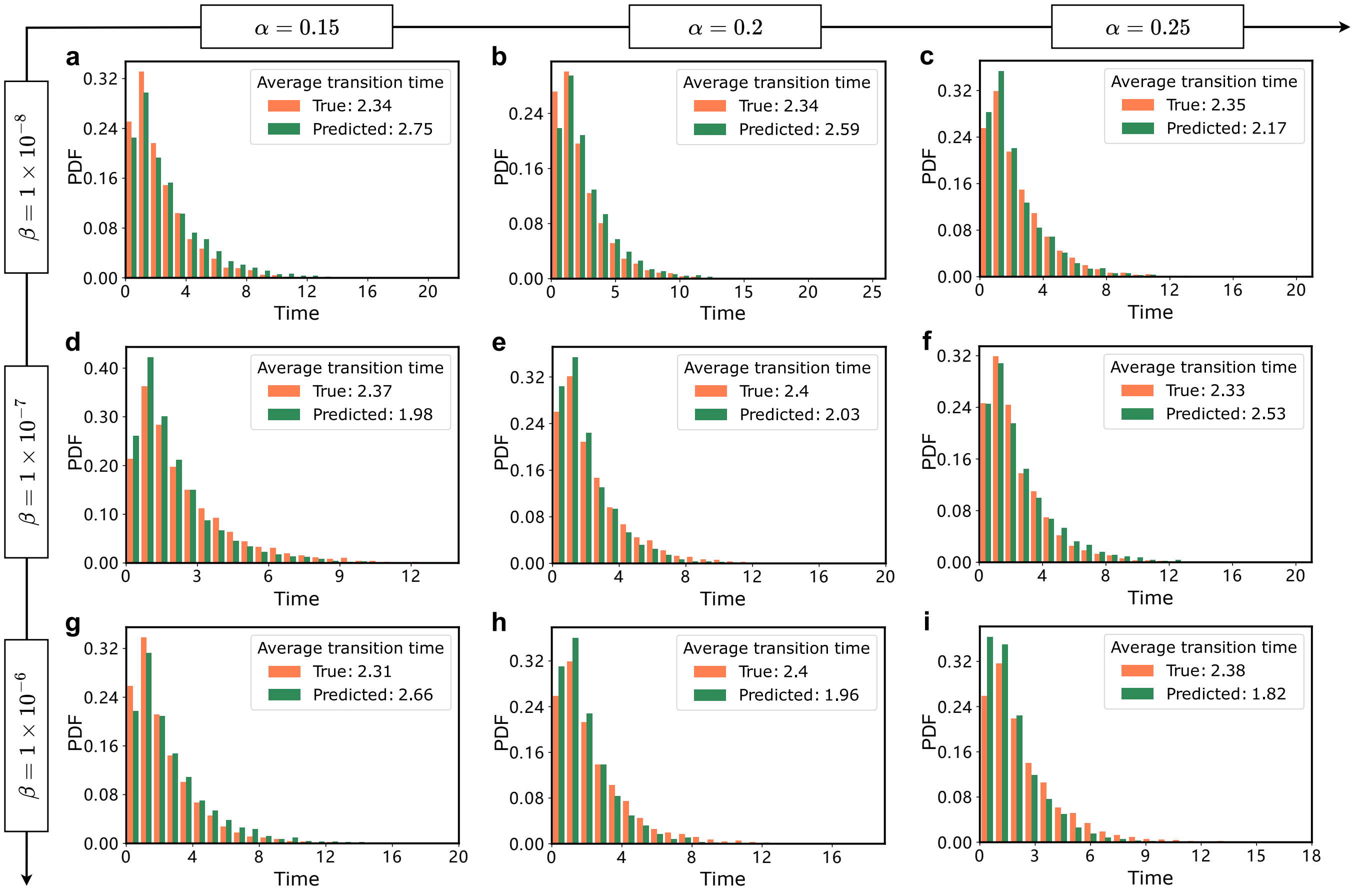

Supplementary FIG. 19. **The robustness of the results in the evaluation under the hyperparameters α and β of the RC.** In the Example 1 of the main text, the hyperparameters $\alpha = 0.2, \beta = 1 \times 10^{-8}$. The length of the predicting set is $10000\delta t$, while $\delta t = 0.01$. PDF: probability density function. (a) Histograms of the transition time for the test and predicted data with $\alpha = 0.15, \beta = 1 \times 10^{-8}$. The results demonstrate the ability to capture stochastic transitions under the values of α and β. (b) Results with $\alpha = 0.2, \beta = 1 \times 10^{-8}$. (c) Results with $\alpha = 0.25, \beta = 1 \times 10^{-8}$. (d-f) The results are the same as in (a-c), with $\beta = 1 \times 10^{-7}$. (g-i) Results same as (a-c), while $\beta = 1 \times 10^{-6}$.

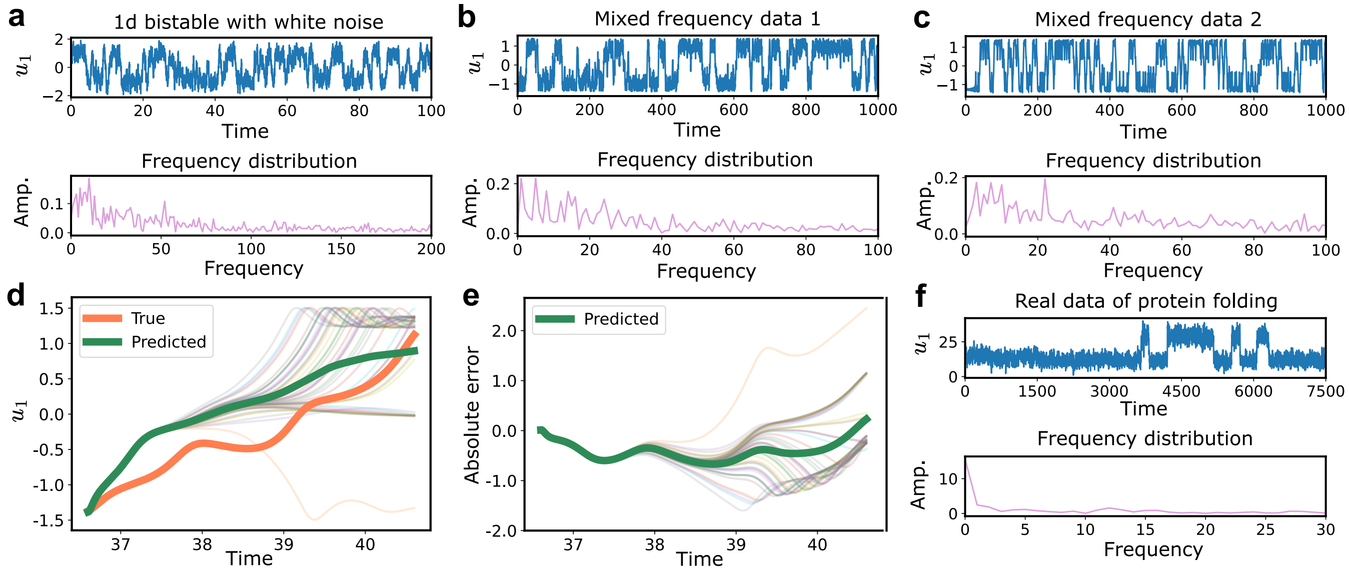

Supplementary FIG. 20. **Results of present method when the frequencies of the time series are mixed.** (a) Upper: generated time series from Eq. (9) of the main text (FIG. 2(b) of the main text). Lower: fast Fourier transform (FFT) result. Amp.: amplitude. (b) Upper: generated time series from Eqs. (10)-(13) of the main text (FIG. 3(b) of the main text). Lower: FFT result. The noise plagues the resonant frequency of the dynamical system of the 1D bistable system. (c) Upper: generated time series (Eqs. (10)-(13) of the main text) by adjusting the parameters of the noise in Eqs. (11)-(13) of the main text ($\epsilon = 1.4, \psi = 0.13$). Lower: FFT result. The frequencies are also mixed. The upper is applied as the training set, and the process is the same as in FIG. 1(a) and FIG. 3(a) of the main text. (d) By repeating the process 50 times with the same hyperparameters, 50 predicted $u_1(t)$ are obtained (fainter lines). The average prediction (thick green) matches the test data (coral). (e) Absolute error of the predicted 50 time series and its mean value (thick green). (f) Upper: experimental data (FIG. 5(a) of the main text). Lower: FFT result of upper.

III. SUPPLEMENTARY TABLES

Supplementary Table I. Identifications of SINDy from data with noise.

SINDy types	Set	threshold	Actual equation	Function
SINDy-2016	1	0.01	$\dot{u}_1 = -5(-u_1 + u_1^3)$	$\dot{u}_1 = -0.0191 - 0.013u_1^3$
SINDy-2016	2	0.005	$\dot{u}_1 = -5(-u_1 + u_1^3)$	$\dot{u}_1 = -0.0111 - 0.01u_1^2 - 0.013u_1^3$
SINDy-2021	3	0.005	$\dot{u}_1 = -5(-u_1 + u_1^3)$	$\dot{u}_1 = -0.0157u_1^2 - 0.0184u_1^3$
SINDy-2021	4	0.001	$\dot{u}_1 = -5(-u_1 + u_1^3)$	$\dot{u}_1 = -0.0039u_1 - 0.0158u_1^2 - 0.0133u_1^3$

Supplementary Table II. The runtime of different approaches.

Approaches	Epoch	Time (second)
RC	1	132
SINDy-2021	3	1043
RNN	10	359

Supplementary Table III. Identifications of SINDy-2016 from filtered data.

Filter types	Q	W	P	Actual equation	Function
Kalman filter	0.05			$\dot{u}_1 = -5(-u_1 + u_1^3)$	$\dot{u}_1 = -0.0161 - 0.014u_1 + 0.013u_1^2 + 0.01u_1^3$
Kalman filter	0.0005			$\dot{u}_1 = -5(-u_1 + u_1^3)$	$\dot{u}_1 = -0.012u_1 + 0.012u_1^3$
Savizky-Golay filter		10	2	$\dot{u}_1 = -5(-u_1 + u_1^3)$	$\dot{u}_1 = -0.0181 - 0.016u_1$
Savizky-Golay filter		50	2	$\dot{u}_1 = -5(-u_1 + u_1^3)$	$\dot{u}_1 = -0.0201 - 0.023u_1 + 0.006u_1^2 + 0.008u_1^3$

Supplementary Table IV. The hyperparameters for section I F 1.

Set	Transitions	δt	T_{train} (Time steps)	T_{predict} (Time steps)	N	K_{in}	D	ρ	α	β
1	Upward	0.01	10000	10000	800	4.2	4	1.1×10^{-3}	0.18	1×10^{-8}
2	Downward	0.01	10000	10000	800	3.8	4	1.2×10^{-3}	0.25	1×10^{-8}

Supplementary Table V. The hyperparameters for section I F 2.

δt	T_{train} (Time steps)	T_{predict} (Time steps)	N	K_{in}	D	ρ	α	β
0.002	20000	20000	1200	1.5	2	1.6×10^{-3}	0.36	1×10^{-7}

Supplementary Table VI. The hyperparameters for section I F 3 and section I F 4.

Set	Transitions	δt	T_{train} (Time steps)	T_{predict} (Time steps)	N	K_{in}	D	ρ	α	β
1	Upward	0.002	20000	20000	1200	1.6	3.6	1.4×10^{-3}	0.58	1×10^{-7}
2	Downward	0.002	20000	20000	1200	1.5	3.5	1.1×10^{-3}	0.64	1×10^{-7}
3	Upward	0.001	25000	25000	1200	1.1	3	9.1×10^{-4}	0.52	1×10^{-7}
4	Downward	0.001	25000	25000	1200	1.3	3	8.9×10^{-4}	0.6	1×10^{-7}

Supplementary Table VII. The hyperparameters for section I F 5.

δt	T_{train} (Time steps)	T_{predict} (Time steps)	N	K_{in}	D	ρ	α	β
0.001	50000	50000	1200	1	2.8	1.7×10^{-3}	0.45	1×10^{-6}

Supplementary Table VIII. The hyperparameters for section I F 6.

Set	δt	T_{train} (Time steps)	T_{predict} (Time steps)	N	K_{in}	D	ρ	α	β
1	0.01	8000	8000	1200	3	3	1.2×10^{-3}	0.45	1×10^{-6}
2	0.01	1000	450	800	0.996	0.996	1	0.048	1×10^{-8}

[1] S. L. Brunton, J. L. Proctor, and J. N. Kutz, Discovering governing equations from data by sparse identification of nonlinear dynamical systems, Proc. Natl Acad. Sci. **113**, 3932 (2016).
[2] A. A. Kaptanoglu, B. M. de Silva, U. Fasel, K. Kaheman, A. J. Goldschmidt, J. L. Callaham, C. B. Delahunt, Z. G. Nicolaou, K. Champion, J.-C. Loiseau, *et al.*, Pysindy: A comprehensive python package for robust sparse system identification, arXiv preprint arXiv:2111.08481 (2021).
[3] K. Kaheman, S. L. Brunton, and J. N. Kutz, Automatic differentiation to simultaneously identify nonlinear dynamics and extract noise probability distributions from data, Mach. Learn.: Sci. Technol. **3**, 015031 (2022).
[4] S. Hochreiter and J. Schmidhuber, Long short-term memory, Neural Comput. **9**, 1735 (1997).
[5] N. K. Manaswi, Understanding and working with keras, in *Deep Learning with Applications Using Python : Chatbots and Face, Object, and Speech Recognition With TensorFlow and Keras* (Apress, Berkeley, CA, 2018) pp. 31–43.
[6] R. R. Labbe, Filterpy documentation (2018).
[7] P. Virtanen, R. Gommers, T. E. Oliphant, M. Haberland, T. Reddy, D. Cournapeau, E. Burovski, P. Peterson, W. Weckesser, J. Bright, *et al.*, Scipy 1.0: fundamental algorithms for scientific computing in python, Nat. Methods **17**, 261 (2020).
[8] D. Sussillo and L. F. Abbott, Generating coherent patterns of activity from chaotic neural networks, Neuron **63**, 544 (2009).
[9] L. B. Liu, A. Losonczy, and Z. Liao, tension: A python package for force learning, PLOS Comput. Biol. **18**, e1010722 (2022).
[10] T. E. Oliphant *et al.*, *Guide to numpy*, Vol. 1 (Trelgol Publishing USA, 2006).
[11] R. Tapia-Rojo, M. Mora, S. Board, J. Walker, R. Boujemaa-Paterski, O. Medalia, and S. Garcia-Manyes, Enhanced statistical sampling reveals microscopic complexity in the talin mechanosensor folding energy landscape, Nat. Phys. **19**, 52 (2023).
[12] D. J. Gauthier, E. Bollt, A. Griffith, and W. A. Barbosa, Next generation reservoir computing, Nat. Commun. **12**, 5564 (2021).
[13] Z. Belkacemi, P. Gkeka, T. Lelièvre, and G. Stoltz, Chasing collective variables using autoencoders and biased trajectories, J. Chem. Theory Comput. **18**, 59 (2021).
[14] S. H. Lim, L. Theo Giorgini, W. Moon, and J. S. Wettlaufer, Predicting critical transitions in multiscale dynamical systems using reservoir computing, Chaos **30**, 123126 (2020).
